# Supplementary material for: Deep Proteome Profiling of White Adipose Tissue Reveals Marked Conservation and Distinct Features Between Different Anatomical Depots
Source: Mol Cell Proteomics. 2023 Feb 12;22(3):100508. doi: 10.1016/j.mcpro.2023.100508 (PMC10014311; doi:10.1016/j.mcpro.2023.100508)
Supplement: Supplemental figure 1 [file mmc1.pdf]

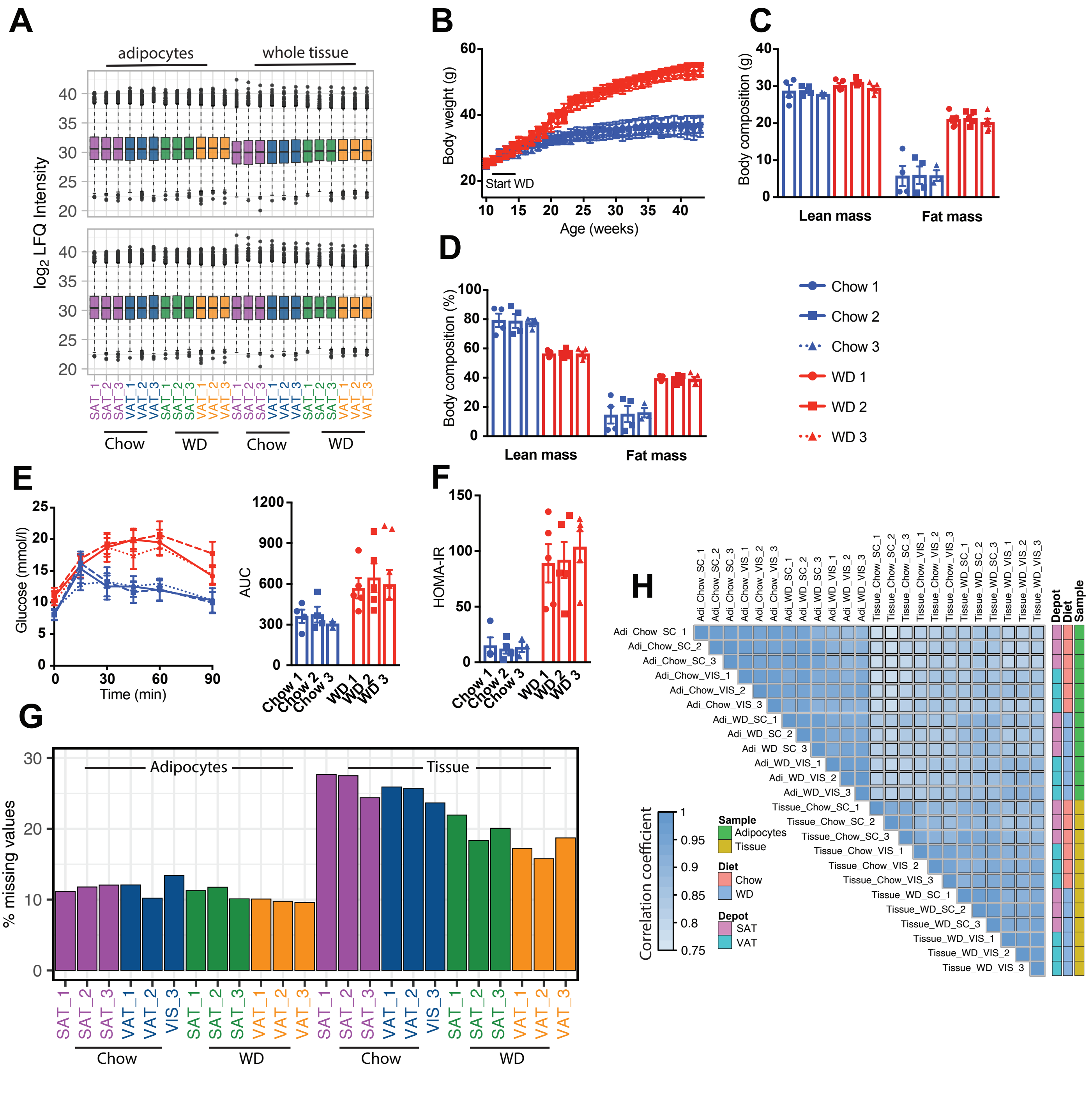

Supplemental Figure 1. (A) Box plot of all proteomics samples before (top) and after (bottom) median normalization. (B) Body weights of mice fed chow of WD for 9 month. (C-D) Body composition in grams (C) and percent (D) at 9 month of diet. (E) Blood glucose and area under the curve during a glucose tolerance test. (F) Homeostatic Model Assessment for Insulin Resistance (HOMA-IR) from fasting blood glucose and plasma insulin levels. (G) Percent missing in proteomics data across all samples. (H) Correlation coefficient for proteomics across all samples.
